# Mapping of p140Cap Phosphorylation Sites: The EPLYA and EGLYA Motifs Have a Key Role in Tyrosine Phosphorylation and Csk Binding, and Are Substrates of the Abl Kinase

**DOI:** 10.1371/journal.pone.0054931

**Published:** 2013-01-31

**Authors:** Daniele Repetto, Simona Aramu, Elisabetta Boeri Erba, Nanaocha Sharma, Silvia Grasso, Isabella Russo, Ole N. Jensen, Sara Cabodi, Emilia Turco, Paola Di Stefano, Paola Defilippi

**Affiliations:** 1 Department of Molecular Biotechnology and Health Sciences, Università degli Studi di Torino, Torino, Italy; 2 Institut de Biologie Structurale JP Ebel, Université J. Fourier, Grenoble, France; 3 Department of Biochemistry and Molecular Biology, University of Southern Denmark, Odense, Denmark; Wayne State University, United States of America

## Abstract

Protein phosphorylation tightly regulates specific binding of effector proteins that control many diverse biological functions of cells (e. g. signaling, migration and proliferation). p140Cap is an adaptor protein, specifically expressed in brain, testis and epithelial cells, that undergoes phosphorylation and tunes its interactions with other regulatory molecules via post-translation modification. In this work, using mass spectrometry, we found that p140Cap is *in vivo* phosphorylated on tyrosine (Y) within the peptide GEGLpYADPYGLLHEGR (from now on referred to as EGLYA) as well as on three serine residues. Consistently, EGLYA has the highest score of *in silico* prediction of p140Cap phosphorylation. To further investigate the p140Cap function, we performed site specific mutagenesis on tyrosines inserted in EGLYA and EPLYA, a second sequence with the same highest score of phosphorylation. The mutant protein, in which both EPLYA/EGLYA tyrosines were converted to phenylalanine, was no longer tyrosine phosphorylated, despite the presence of other tyrosine residues in p140Cap sequence. Moreover, this mutant lost its ability to bind the C-terminal Src kinase (Csk), previously shown to interact with p140Cap by Far Western analysis. In addition, we found that in vitro and in HEK-293 cells, the Abelson kinase is the major kinase involved in p140Cap tyrosine phosphorylation on the EPLYA and EGLYA sequences. Overall, these data represent an original attempt to in vivo characterise phosphorylated residues of p140Cap. Elucidating the function of p140Cap will provide novel insights into its biological activity not only in normal cells, but also in tumors.

## Introduction

p140Cap encoded by the Srcin1 gene, is a docking protein specifically expressed in brain, testis and epithelial cells [1]–[5]. So far p140Cap has been mostly studied in epithelial tumor cells, where it regulates integrin and growth factor-dependent carcinoma cell properties, involved in tumor progression [5]–[8]. In addition p140Cap has been analyzed in neurons, where it can control synapse formation/maintenance [1], [3], [4].

p140Cap is composed of a tyrosine-rich domain, two proline-rich regions, a coil-coiled domain, two regions rich in charged amino acids and a putative actin binding site [2]. Several of these conserved domains have been already shown to associate with specific partners. In particular p140Cap was originally identified to bind through coil-coiled interactions to the synaptic membrane protein SNAP-25 [1]–[5], and, through its second proline-rich region, to Src kinase [6], Vinexin [3], and Cortactin [8]. Moreover, the C-terminal domain of p140Cap associates to EB3, a member of the microtubule plus-end tracking protein EB family [4].

p140Cap contains several serine and tyrosine residues, which could undergo phosphorylation upon different biological stimuli. Using large-scale phosphoproteomic studies ([9] and http://www.phosphosite.org/), p140Cap phosphorylation sites have been identified in distinct cell lines, but their role has not been characterised. We have already shown that p140Cap is tyrosine phosphorylated in epithelial cells upon integrin-mediated adhesion and EGF treatment [2]. However, elucidating the functional interplay between multiple p140Cap phosphorylated residues and their role as binding sites remains a major challenge.

Csk and the Csk-homologous kinase (Chk) are endogenous inhibitors constraining the activity of the Src-family kinases (SFKs) in cells. Both kinases suppress SFKs by selectively phosphorylating their consensus C-terminal regulatory tyrosine [10], [11]. We have previously shown that, upon cell-extracellular matrix adhesion or EGF stimulation, p140Cap activates Csk. This kinase phosphorylates an inhibitory tyrosine on the C-terminal domain of Src allowing the closure of Src in an inactive conformation [6]. Although we have already shown that Csk directly interacts with p140Cap [6], the nature of this interaction has not been fully elucidated.

Mass spectrometry (MS)-based proteomics has been widely used for studies of protein phosphorylation [12]. It has been significantly improved by phosphorylation-directed multistage tandem MS (pdMS^3^) using liquid chromatographic separation (LC) and hybrid linear ion trap (LTQ)-FT mass spectrometers [13]. This approach allows the accurate measurement of parent ion masses, by a Fourier transform ion cyclotron resonance (FTICR) “selected ion monitoring” (SIM) scan, and the detection of diagnostic neutral loss of phosphoric acid (98 Da). This diagnostic loss from the precursor ion, detected in a MS^2^ mode automatically triggered data-dependent MS^3^ fragmentation of the precursor ion. This results in high yield of peptide backbone fragments, determining a high confidence peptide identification and phosphorylation site assignment [14].

Here we applied pdMS^3^-based analytical strategy targeted toward the detection and sequencing of *in vivo* phosphopeptides derived from p140Cap and we identified one phosphotyrosine and three phosphoserine residues. By site directed mutagenesis we defined the tyrosines contained in the sequences EGLYA and EPLYA as the major residues responsible for p140Cap tyrosine phosphorylation and functionally validated their essential role in binding to the Csk kinase. Moreover, we found that *in vitro* an in Human Embryonic Kidney (HEK)-293 cells, the Abelson (Abl) kinase is the major tyrosine kinase able to trigger p140Cap phosphorylation on these motifs.

## Materials and Methods

### Cell Lines, Reagents, Antibodies

Human embryonic kidney HEK-293 and human breast cancer MCF7 cells were obtained from ATCC and cultured in DMEM with 10% FCS and Penicillin/Streptomycin. p140Cap monoclonal and polyclonal antibodies were produced in our laboratory as previously described in [2]. Antibodies to Src, Vinculin, Csk, and phosphotyrosine PY99 were from Santa Cruz Biotechnology (Santa Cruz, CA), to GST from BD Bioscience (2350 Qume Drive San Jose, CA 95131), and to Tubulin from SIGMA, to Glutathione-Sepharose, Protein A-Sepharose, Protein G-Sepharose, Horse radish peroxidase, nitrocellulose and films were from GE Healthcare Bio-Science AB. Tissue culture media, serum and antibiotics were from Invitrogen. Src kinase specific inhibitor SU6656 was obtained from SIGMA. Abl kinase specific inhibitor (Imatinib) was a kind gift of Dr. Giuseppe Saglio (University of Torino).

### Mass Spectrometry

Nanoscale liquid chromatography mass spectrometry experiments were performed on an Agilent 1100 nanoflow system (Agilent Technologies) coupled a 7-tesla Finnigan LTQ-FT mass spectrometer (Thermo Electron, Bremen, Germany) equipped with a nanoelectrospray ion source (Proxeon Biosystems, Odense, Denmark) as described previously [14]. The following instrumental conditions were used: spray voltage, 2.4 kV; no sheath and auxiliary gas flow; ion transfer tube temperature, 100°C; collision gas pressure, 1.3 millitorrs. A data-dependent mode allowed the LTQ-FT mass spectrometer to automatically switch between MS, MS^2^, and neutral loss-dependent MS^3^ acquisition. Survey full scan MS spectra were acquired between m/z 300 Th and 1600 Th by FTICR with resolution r 25000 at m/z 400. Then, the three most intense ions of each survey full scan MS spectrum were sequentially isolated for accurate mass measurements by a FTICR “selected ion monitoring” (SIM) scan. These ions were also fragmented in the linear ion trap using collision-induced dissociation. Data-dependent settings were enabled to trigger a MS^3^ scan when a neutral loss of 97.99, 48.99 or 32.66 Da (singly, doubly and triply charged phosphopeptides) was detected among the 10 most intense fragment ions of the MS^2^ spectra.

### Database Searching

MS^n^ data were processed (smoothing, background subtraction, and centroiding) using the program DTASuperCharge (Source- Forge, Inc.). The processed files were subsequently searched against the human sequence library in the International Protein Index (IPI) and UniProtKB/Swiss-Prot protein sequence databases using an in-house Mascot server (Matrix Science Ltd.,London, UK). The databases searches were performed choosing trypsin as enzyme and one miss cleavage was allowed. Carbamidomethyl (Cys) was considered as the fixed modification. Oxidation (Met), N-acetylation (protein) and phosphorylation (STY) were chosen as variable modifications. The data were searched with a peptide mass tolerance of ±30 ppm and a fragment mass tolerance of ±0.6 Da.

### cDNA Constructs and Site Specific Mutagenesis of p140Cap

pEGFP-N1-p140Cap cDNA [2] was used to generate single, double or triple point mutation of p140Cap tyrosines into phenylalanine (Y/F) following manufacture instruction of Quickchange Lightening Mutagenesis kit (Agilent Technologies, Inc.Life Sciences and Chemical Analysis Group Santa Clara, CA 95051-7201USA), and using the primers:

EPLY/FA F 5′GAAGGAGCCGTTGTTTGCTGCTTTTCCTGGC

EPLY/FA R 5′GCCAGGAAAAGCAGCAAACAACGGCTCCTTC

EGLY/FA F 5′GGGCGAGGGCCTCTTTGCCGATCCCTACGGG

EGLY/FA R 5′CCCGTAGGGATCGGCAAAGAGGCCCTCGCCC

FY/FELE F 5′GCTCGCAATGTCTTCTTCGAGCTGGAAGACGTC

FY/FELE R 5′GACGTCTTCCAGCTCGAAGAAGACATTGCGAGC

DPY/FG F 5′TGCCGATCCCTTCGGGCTGCTGCACGAGG

DPY/FG R 5′CCTCGTGCAGCAGCCCGAAGGGATCGGCA.

pcDNA3-BCR-Abl coding for the active form of Abl [15] was a kind gift of Prof. G. Saglio, University of Torino.

### Production of Recombinant Csk Mutants

Csk full length was cloned into pQE vector by amplification of I.M.A.G.E. full Length cDNA clone IRAVp968G01111D, using specific primers. The Csk SH3 (37–182 bp from ATG) and SH2 (239–513 bp from ATG) domains were amplified by Csk full length using specific primers, and the amplification products were cloned into pGEX 4T3 vector using BamHI and EcoRI restriction enzymes. Csk Delta SH2 was originated by cloning in the pQE vector two fragments, one amplified from 1 to 239 bp with BamHI and XbaI restriction sites at the end, and the other amplified from 513 to the stop codon with XbaI and HindIII restriction sites at the end. Recombinant fusion proteins were produced in E. coli as described [2].

### Far Western

Far-Western analysis was performed as described in [6]. Briefly, membranes were blocked for 1 h in TBS, 0.1% Tween-20, 5% BSA and incubated for 3 h with 1 microgram/ml of different Csk recombinant proteins. Filters were first decorated with anti-Csk or anti GST antibody and subsequently with anti-p140Cap.

### Biochemical Analysis of p140Cap Tyrosine Phosphorylation

To analyze phosphorylation of p140Cap (NCBI Reference Sequence: NP_079524.2; REFSEQ: accession NM_025248.2) and of its mutants, HEK-293 cells were transiently transfected with the corresponding cDNAs by conventional calcium phosphate precipitation protocols. 48 hrs after transfection, cells were extracted with lysis buffer (50 mM HEPES (pH 7.5), 150 mM NaCl, 1.5 mM MgCl_2_, 1 mM EDTA, 1% Triton X-100, and 10% glycerol) containing protease and phosphatase inhibitors (1 mM PMSF, 10 microgram/ml leupeptin, 10 microgram/ml aprotinin, 10 mM NaF, and 1 mM Na_3_VO_4_). In some experiments cells were treated for 5 minutes with 100 micromolar of Pervanadate Stock solution (30 mM of Sodium ortovanadate and 0,18% Hydrogen peroxide diluted in PBS 1X) or starved overnight and stimulated with 20% Fetal Bovine Serum (FBS) for 30 min. In specific experiments cells were transfected with p140Cap and its mutant together with the pcDNA-3-BCR-Abl construct. Cell extracts were centrifuged at 13,000×*g* for 10 minutes, and the supernatants were collected and assayed for protein concentration using the Bio-Rad protein assay method (Bio-Rad). For immunoprecipitation, Dynabeads (Invitrogen) were cross linked with the specific p140Cap monoclonal antibody, following manufacture protocol. Cell extracts were incubated with the Dynabeads-conjugated antibody overnight at 4°C. The immunoprecipitates were washed three times with 1 ml of lysis buffer, resolved on SDS-PAGE, transferred onto nitrocellulose and reacted with specific antibodies. As input, fifty micrograms of cell extracts were analysed by SDS-PAGE and Western Blotting.

### Identification of Tyrosine Kinase Activity on Synthetic EPLYA and EGLYA Peptides

This assay was performed by ProQinase (Freiburg, GERMANY). The phosphorylation profile of two biotinylated sample peptides, SIIKIYRKEPLYAAFPGSHLTNGDL and LAGKAGGMVLVKGEGLYADPYGLLH, synthesized by JPT Peptide Technologies (Berlin, Germany) was determined at three concentrations each (1, 0.5, 0.25 micromolar) in triplicate in a radiometric activity assay based on streptavidin-coated FlashPlateTM Plus, using three recombinant protein kinases, Abl, Alk and Src, respectively.

The reaction cocktails were pipetted into 96 well, V-shaped polypropylene microtiter plates (“assay plates”) in the following order: 10 microliters of kinase solution, 40 microliters of buffer/ATP/test sample mixture. The reaction cocktails contained 60 mM HEPES-NaOH, pH 7.5, 3 mM MgCl_2_, 3 mM MnCl_2_, 3 micromolar Na_3_VO_4_, 1.2 mM DTT, 50 microgram/ml PEG20000, 1 micromolar ATP/[gamma P^33^]-ATP (8.0×1005 cpm per well), protein kinase and sample peptide (1/0.5/0.25 micromolar). The assay plates were incubated at 30°C for 60 minutes. Subsequently, the reaction cocktails were stopped with 20 microliters of 4.7 M NaCl/35 mM EDTA. The reaction cocktails were transferred into 96-well streptavidin-coated FlashPlateTM Plus (PerkinElmer, Boston, MA, USA), followed by 30 min incubation at room temperature on a shaker. Subsequently the plates were aspirated and washed three times with 250 microliters of 0.9% NaCl. Kinase activity dependant transfer of P^33^ (“counting of cpm”) was determined with a microplate scintillation counter (Microbeta, Perkin Elmer).

## Results

### *In vivo* Mapping of p140Cap Phosphorylation Sites

**Figure 1:**
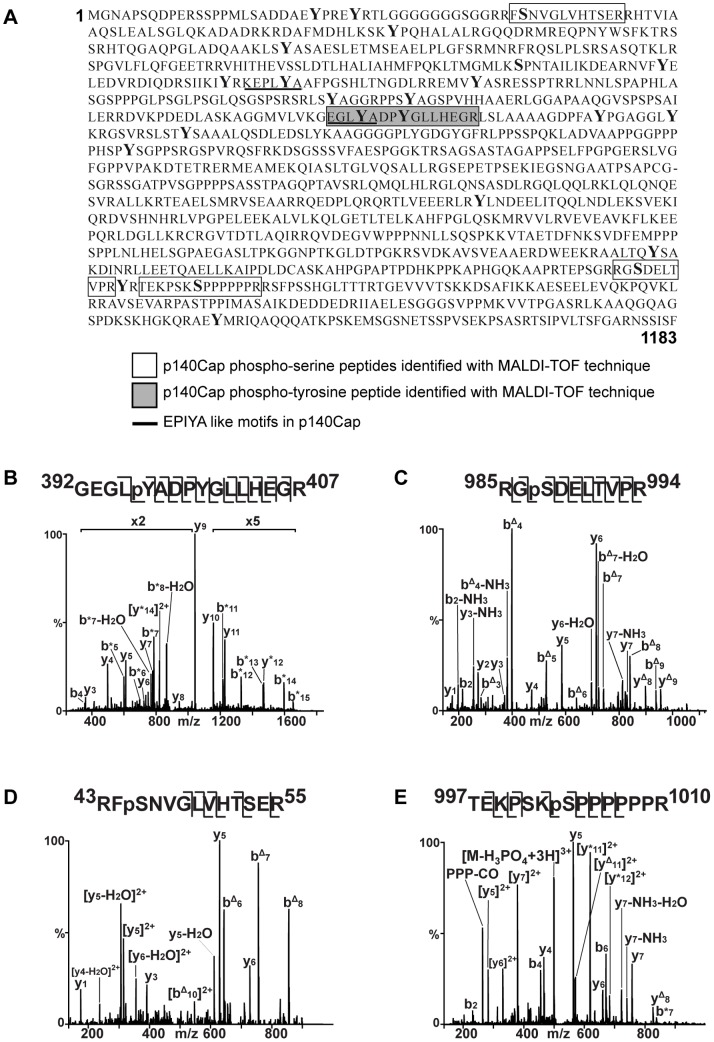
Amino acid sequence of p140Cap and spectra of *in vivo* phosphorylated peptides. A. Amino acid sequence of p140Cap (NCBI Reference Sequence: NP_079524.2; REFSEQ: accession NM_025248.2) showing, the peptides found to be phosphorylated by MS analysis (phosphoserine: blank open box; phosphotyrosine: grey box) and the EPIYA-like motifs (underlined). All the tyrosine residues present in p140Cap are indicated in Bold. Note that the phosphoserines in position 45, 987 and 1003 are conserved among human and murine sequences. **B.** The phosphopeptide 392-GEGLpYADPYGLLHEGR-407 with m/z 913.9091 (z: +2) was sequenced by LC-MS^2^. The signals of ions within 350–1030 and 1100–1685 m/z were enhanced of 2 and 5 times, respectively. The signal enhancement facilitated the labeling of the ions. Only the most relevant fragment ion signals are labeled in the MS^2^ spectrum. *, Ions containing phosphorylated tyrosine-396 (pY396). **C.** MS^3^ spectrum of the phosphopeptide 985-RGpSDELTVPR-994. In MS^2^ mode the parent ion at m/z 605.2843 Th (z: 2+) lost phosphoric acid generating an ion at m/z 556.2868 Th, which was subjected to MS^3^ fragmentation. Delta indicates the loss of phosphoric acid. **D.** MS^3^ spectrum of the peptide 43-RFpSNVGLVHTSER-55. **E.** MS^2^ spectrum of 997-TEKPSKpSPPPPPPR-1010. *, Ions containing phosphorylated serine-1003 (pS1003). PPP-CO indicates an internal fragment ion containing three proline residues which has lost carbon monoxide (28 Da).

p140Cap docking protein includes a number of serine and tyrosine residues (Figure 1A) that upon phosphorylation could serve as binding sites for other proteins. We used pdMS^3^ approach to detect the *in vivo* phosphorylation of p140Cap in human breast cancer cells. p140Cap was purified from extracts of MCF7 cells by immunoprecipitation with specific monoclonal antibodies against p140Cap, and subjected to tryptic digestion. The MS and computational analyses confirmed that the protein recovered from the SDS-PAGE gel band contained only the p140Cap protein. They also revealed four singly phosphorylated peptides with masses 1208.5540, 1580.7613, 1593.7919 and 1825.9329 Da (Table 1).

**Table 1:** Phosphopeptides of p140Cap protein.

	M (Da)	p140Cap phosphopeptides sequence
**Ser 45**	1580.7613	43-RFpSNVGLVHTSER-55
**Ser 987**	1208.5540	985-RGpSDELTVPR-994
**Ser 1003**	1593.7919	997-TEKPSKpSPPPPPPR-1010
**Tyr 396**	1825.9329	392-GEGLpYADPYGLLHEGR-407

*In vivo* phosphorylated p140Cap was immunoprecipitated from MCF7 human breast cancer cells and four phosphopeptides were recovered by liquid chromatography, analysed by mass spectrometry (MS) and subjected to MS^2^ and MS^3^ fragmentation. We indicate the position of phosphorylation sites within the NCBI Reference sequence NM_025248.2, the masses of the peptides (expressed in Da) and their amino acidic sequence.

Manual interpretation of the MS^2^ and MS^3^ spectra allowed us to confidently allocate the position of the phosphorylated sites of p140Cap. The MS^2^ spectrum of the peptide with mass 1825.8036 Da [mass/charges (m/z): 913.9091 Th, charges (z): 2+] showed an extensive peptide backbone fragmentation, allowing a high-confidence phosphorylation assignment (Figure 1B). In particular, amino-terminal b_5_, b_6_, b_7_ and b_8_ ions indicated that the phosphorylation was located on tyrosine-396. Furthermore, carboxyl terminal y-ions (i.e. y_3_, y_4_, y_5_, y_6_, y_7_, y_8_, y_9_, y_10_, y_11_ ions) did not contain a phosphoryl group and ruled out the possibility of phosphotyrosine-400. Therefore, the *in vivo* mapping revealed the presence of a tyrosine phosphorylated residue, inserted in the sequence 392-GEGLpYADPYGLLHEGR-407 (briefly indicated as EGLYA) (Table 1, and Figure 1B). Interestingly the tyrosine included in the EGLYA sequence has also been found phosphorylated by a large-scale identification of tyrosine phosphorylation sites from murine brain [16].

In MS^2^ spectrum the peptide with mass 1208.5540 Da, (m/z: 605.2843 Th, z: 2+) lost 48.9975 Th generating an ion (m/z: 556.2868 Th, z: 2+) which was subjected to MS^3^ analysis (Figure 1C). The fragmentation pattern showed that serine-987 was phosphorylated (i.e. RGpSDELTVPR). The peptide 43-RFpSNVGLVHTSER-55 (mass: 1580.7614 Da; m/z: 527.9277 Th, z: 3+) was subjected to MS^2^ and MS^3^ fragmentation (Figure 1D), indicating that serine-45 was phosphorylated (Figure 1D). The MS^2^ spectrum of the peptide 997-TEKPSKpSPPPPPPR-1010, whose mass is 1593.7919 Da (m/z 532.2713 Th, z: 3+), is shown in Figure 1E. The presence of phosphorylated b_7_ ion (indicated as b*_7_) and of y_8,_ which lost phosphoric acid (y^Delta^_8_) suggested the presence of the phosphorylated serine-1003. The b_2_, b_4_, b_6_ ions are not modified, excluding the presence of a phosphoryl group on threonine-997 or serine-1001. Overall, three peptides contained phosphorylated serine residues (Figure 1C–E), namely RGpSDELTVPR, RFpSNVGLVHTSER, and TEKPSKpSPPPPPPR were identified. Among them, serine S45 and S987 are conserved among human and mouse p140Cap sequences, and have been previously identified in murine phosphoproteomic screens (http://www.uniprot.org/uniprot/Q9QWI6), underlying the relevance of phosphorylation of these sites across the species.

In human breast cancer cells p140Cap undergoes *in vivo* phosphorylation on multiple sites, specifically identified in one tyrosine and three serine residues. Since we have shown that integrin-mediated adhesion or serum treatment induce p140Cap tyrosine phosphorylation [2] we focused our attention on tyrosine residues to evaluate the function of Y396 in the p140Cap protein.

### p140Cap Tyrosine Phosphorylation Relies on Two Major Tyrosine Residues Included in the Sequences EGLYA and EPLYA

Since we have already demonstrated that p140Cap directly binds to Csk [6], we were interested in assessing the relevance of tyrosine phosphorylation of the EGLYA motif in Csk binding. Interestingly, p140Cap also contains a sequence analogous to EGLYA, represented by the EPLYA motif (see Figure 1A). These two sequences are similar to the EPIYA motif, previously found in the bacterial CagA protein involved in Helicobacter Pylori pathogenesis [17]–[20], and in the mammalian Pragmin/SgK223 [21]. Upon tyrosine phosphorylation at the EPIYA motif, these proteins acquire the ability to interact with the SH2 domain of Csk.

We closely examined our liquid chromatography data in order to find evidence of the in vivo Y264 phosphorylation in MCF7 cells. The non-phosphorylated form of the peptide 261-EPLYAAFPGSHLTNGDLR-278 was automatically subjected to MS/MS fragmentation. This peptide (mass: 1956.96 Da) was detected as 3+ (m/z: 653.33), at retention time (RT) 30.17 min (data not shown). Thanking into account these features, we calculated a possible mass of the 261–278 phosphorylated form (2036.93 Da). In a chromatogram at RT 30.46 min, there was peak at m/z 679.99, (z: 3+), indicating a mass of 2036.93. This could correspond to the phosphorylated 261–278 peptide, but it was not selected for MS/MS fragmentation. In other runs we did not find the 679.99 peak suggesting that the tyrosine 264 residue (EPLYA) can be phosphorylated in a very dynamic way whose kinetics is different from the tyrosine 396 (EGLYA) in human breast cancer MCF7 cells (data not shown).

**Figure 2:**
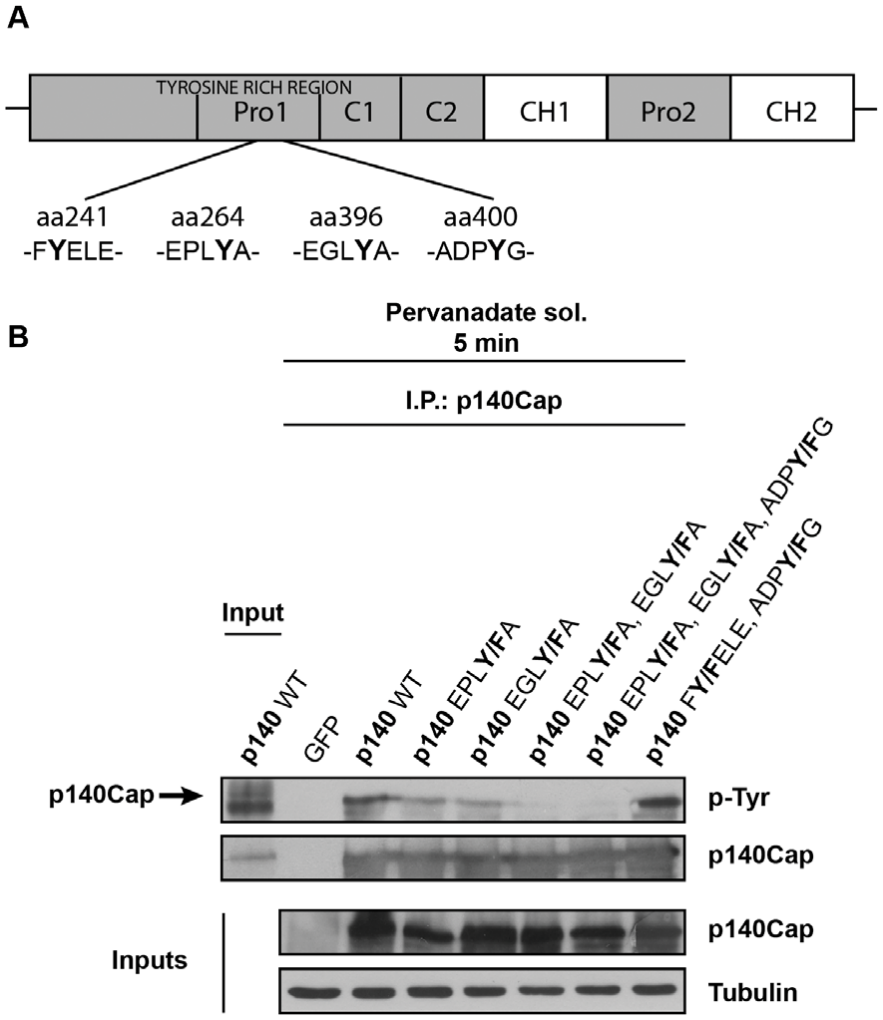
p140Cap tyrosine phosphorylation depends mainly on two tyrosine residues. A. Schematic representation of p140Cap structure and localization of FYELE, EPLYA, EGLYA, and ADPYG sequences into the tyrosine rich region. These four tyrosine residues have been mutated to phenylalanine. **B.** cDNAs encoding GFP, GFP-p140Cap full length (p140 WT) and its single (p140 EPL**Y/F**A, p140 EGL**Y/F**A), double (p140 EPL**Y/F**A, EGL**Y/F**A; p140 F**Y/F**ELE, ADP**Y**/**F**G) and triple (p140 EPL**Y/F**A, EGL**Y/F**A, ADP**Y**/**F**G) mutants were used to transfect HEK-293 cells. 48 hours after transfection cells were treated for 5 minutes with 100 micromolar pervanadate solution. Cell extracts were immunoprecipitated with a specific antibody to p140Cap and immunocomplexes were analysed by western blotting using monoclonal antibodies to phosphotyrosine (PY99), p140Cap and Tubulin respectively. The results are representative of six independent experiments.

Taking into account our *in vivo* data, we used the NetPhos program to predict the score of putative phosphorylation by tyrosine kinases [22]. The EGLYA and EPLYA tyrosines showed the highest probability score (0.981 and 0.980, respectively, Table 2), leading us to perform site-specific mutagenesis to convert these two tyrosines to phenylalanine. We also selected two low score tyrosines, one inserted in the peptide GEGLpYADPYGLLHEGR (ADPYG) and the other in the peptide RNVFYELED (FYELE) as negative internal controls (Figure 2A).

**Table 2:** Prediction of tyrosine phosphorylation and relative score.

Pos	Context	Score
24	DDAEYPREY	0.572
28	YPREYRTLG	0.104
91	LKSKYPQHA	0.696
111	EQPNYWSFK	0.884
138	AKLSYASAE	0.818
241	RNVFYELED	0.220
258	IIKIYRKEP	0.008
264	KEPLYAAFP	0.980
283	REMVYASRE	0.523
332	SRLSYAGGR	0.235
340	RPPSYAGSP	0.875
396	GEGLYADPY	0.981
400	ADPYGLLH	0.400
420	DPFAYPGAG	0.137
427	AGGLYKRGS	0.206
438	SLSTYSAAA	0.379
452	EDSLYKAAG	0.952
462	GGPLYGDGY	0.683
466	GDGYGFRL	0.488
495	PHSPYSGPP	0.353
722	ERLRYLNDE	0.342
995	TVPRYRTEK	0.049
1131	QRAEYMRIQ	0.339

The human p140Cap protein was analysed with the NetPhos algorithm [22] to obtain a score of putative phosphorylation for each of the 24 tyrosines present in the sequence. Pos. indicates the first tyrosine position in the human p140Cap protein. Context identifies the amino acid sequence surrounding each tyrosine.

Wild type p140Cap (WT) and the mutant cDNAs were transiently transfected in Human Embryonic Kidney 293 (HEK-293) cells. Within 48 hours upon transfection, cells were treated with 100 micromolar pervanadate solution for 5 minutes to significantly inhibit phosphatase action and enhance tyrosine phosphorylation. p140Cap and its mutants were then immunoprecipitated and analysed for tyrosine phosphorylation by blotting with antibodies to phosphorylated tyrosines. In cells expressing the single tyrosine mutant EPL**Y/F**A or EGL**Y/F**A, p140Cap tyrosine phosphorylation was decreased compared to WT, indicating that both tyrosine residues contribute to p140Cap phosphorylation. Strikingly, the double mutant (EPL**Y/F**A, EGL**Y/F**A) was completely defective in phosphorylation (Figure 2B). In contrast, the single mutants on F**Y/F**ELE or ADP**Y/F**G (not shown) and the double mutant (F**Y/F**ELE, ADP**Y/F**G) were still phosphorylated on tyrosine at a similar extent of the WT (Figure 2B). Therefore, the tyrosines inserted in the sequences EPLYA and EGLYA are the major p140Cap phosphorylated sites.

### p140Cap and Csk Directly Associate through the SH2 Domain of Csk

**Figure 3:**
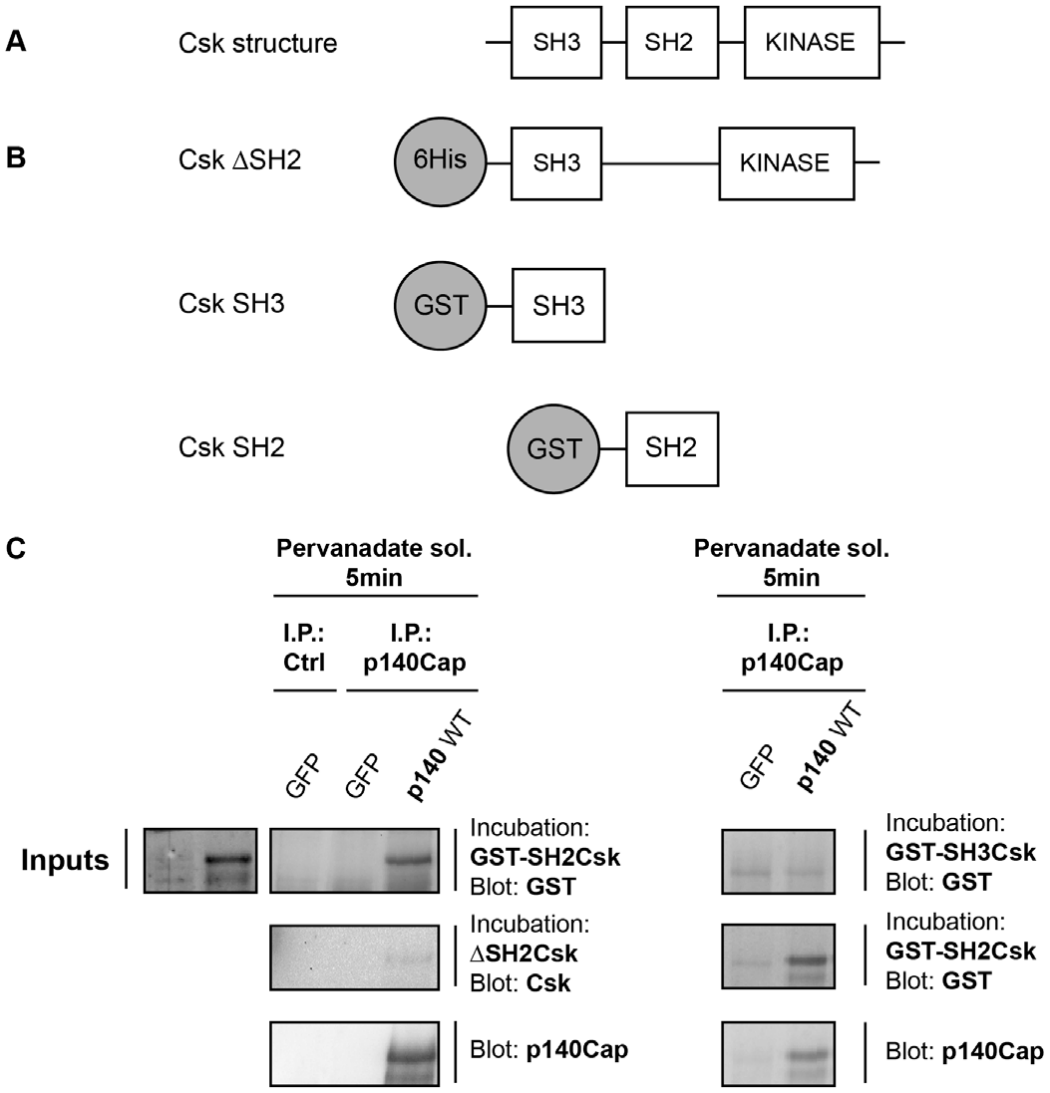
p140Cap binds directly to Csk through Csk SH2 domain. A. A schematic representation of full length Csk kinase protein domains. **B.** A schematic representation of different Csk recombinant mutant proteins. **C.** HEK-293 cells transfected with GFP or GFP-p140Cap full length (p140 WT) were treated with 100 micromolar pervanadate solution as in Figure 2B. Cell extracts were immunoprecipitated with a specific antibody to p140Cap or a pre immune serum as negative control (Ctrl). Immunocomplexes run on 6% SDS-PAGE and transferred to nitrocellulose, were analysed by Far western blotting, incubating with the different Csk recombinant proteins and probing with antibodies specific for GST, Csk and p140Cap. The results are representative of three independent experiments.

Our previous results showed that p140Cap can directly interact with the Csk protein by Far western analysis [6]. In addition to its catalytic domain, Csk contains both a SH2 and a SH3 domain (Figure 3A). To assess how Csk can mediate binding to p140Cap, bacterial recombinant GST fusion proteins expressing Csk SH2 or SH3 domains, were produced. Moreover, a bacterial fusion protein expressing a Csk mutant deleted of the SH2 domain (Csk DeltaSH2) was also generated (Figure 3B). HEK-293 cells were transiently transfected with p140Cap cDNA and within 48 hours upon transfection p140Cap was immunoprecipitated, run on a SDS-PAGE and subjected to Far Western blotting by incubation with Csk recombinant mutant proteins. Figure 3C shows that only the Csk SH2 domain was able to bind p140Cap, while the Csk DeltaSH2 mutant did not associate. Consistently, the SH3 domain of Csk was not able to bind p140Cap (Figure 3C). Overall these results indicate that p140Cap and Csk directly interact through the SH2 domain of Csk.

### The EPLYA and EGLYA Tyrosines Mediate Phosphorylation-dependent Csk Binding to p140Cap

**Figure 4:**
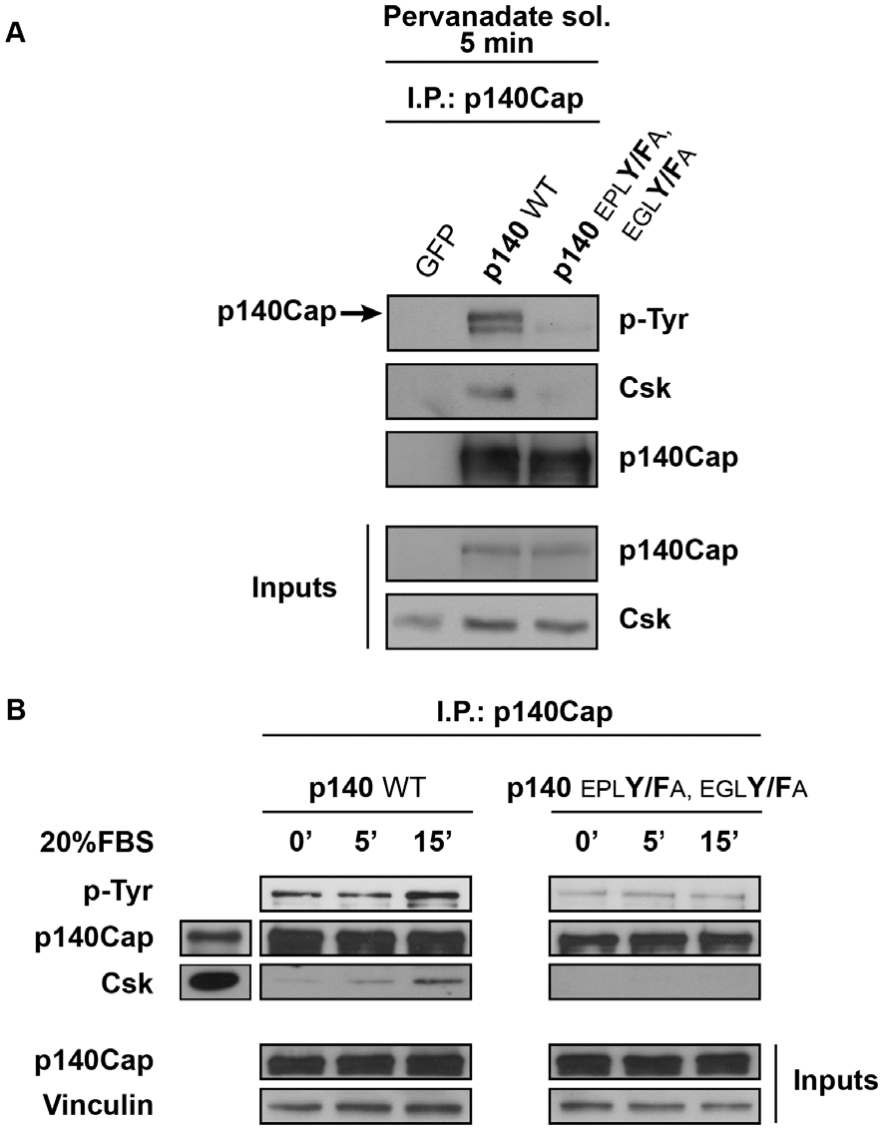
p140Cap tyrosine phosphorylation on EPLYA and EGLYA sequences regulate Csk binding. **A.** cDNAs encoding GFP, GFP-p140Cap full length (p140 WT) and its double mutant (p140 EPL**Y/F**A, EGLY**/F**A) were used to transfect HEK-293 cells. Cells were treated with 100 micromolar pervanadate solution as in Figure 2B and extracts were immunoprecipitated with a specific antibody to p140Cap and analysed by western blotting using monoclonal antibodies PY99, p140Cap, and Csk. The results are representative of six independent experiments. **B.** HEK-293 cells transfected as in A for 48 hours, were starved overnight and treated for 0, 5, 15 minutes with 20%FBS. Cell extracts were immunoprecipitated with a specific antibody to p140Cap. Immunocomplexes were analysed by western blotting using monoclonal antibodies specific for phosphotyrosines, p140Cap, Csk and Vinculin.

Tyrosine phosphorylation at the EPIYA-like motif allows the protein CagA and Pragmin/Sgk223 to interact with the SH2 domain of Csk [20], [21]. To assess the relevance of EPLYA and EGLYA phosphorylated tyrosines to p140Cap-Csk interaction, transiently transfected HEK-293 cells were treated with 100 micromolar pervanadate solution for 5 minutes. The presence of Csk was evaluated in the immunoprecipitates of WT and p140Cap mutants by western blot. As shown in Figure 4A, while Csk was detected in the immunoprecipitate obtained from WT transfected cells, it was absent in those deriving from the extracts of cells transfected with the double p140Cap mutant (EPL**Y/F**A, EGL**Y/F**A), indicating that these two phosphorylated tyrosines mediate Csk binding.

To further investigate p140Cap-Csk binding upon tyrosine phosphorylation induced by a physiological stimulus, starved cells were treated with 20% FBS for different times. As shown in Figure 4B, p140Cap tyrosine phosphorylation was induced over a basal level within 15 minutes of FBS treatment, with a parallel increase in Csk binding (left panels). In cells transfected with the EPL**Y/F**A, EGL**Y/F**A double mutant, upon FBS treatment, p140Cap tyrosine phosphorylation did not increase over the basal level that was already much lower than that of p140Cap WT. In these conditions Csk was not detectable in p140Cap EPL**Y/F**A, EGL**Y/F**A double mutant immunoprecipitates (Figure 4B, right panels). Accordingly, while Src kinase activity, reported as phosphorylation of Y416, was inhibited in cells expressing p140Cap WT; it was partially rescued by over-expression of the EPL**Y**/**F**A, EGL**Y**/**F**A double mutant (Supplementary Figure S1). In summary, Csk associates only to tyrosine phosphorylated p140Cap and upon phosphorylation the tyrosine residues EPLYA and EGLYA mediate the binding to Csk, resulting in inhibition of Src kinase activity.

### p140Cap is a Substrate of the Abelson Tyrosine Kinase

**Figure 5:**
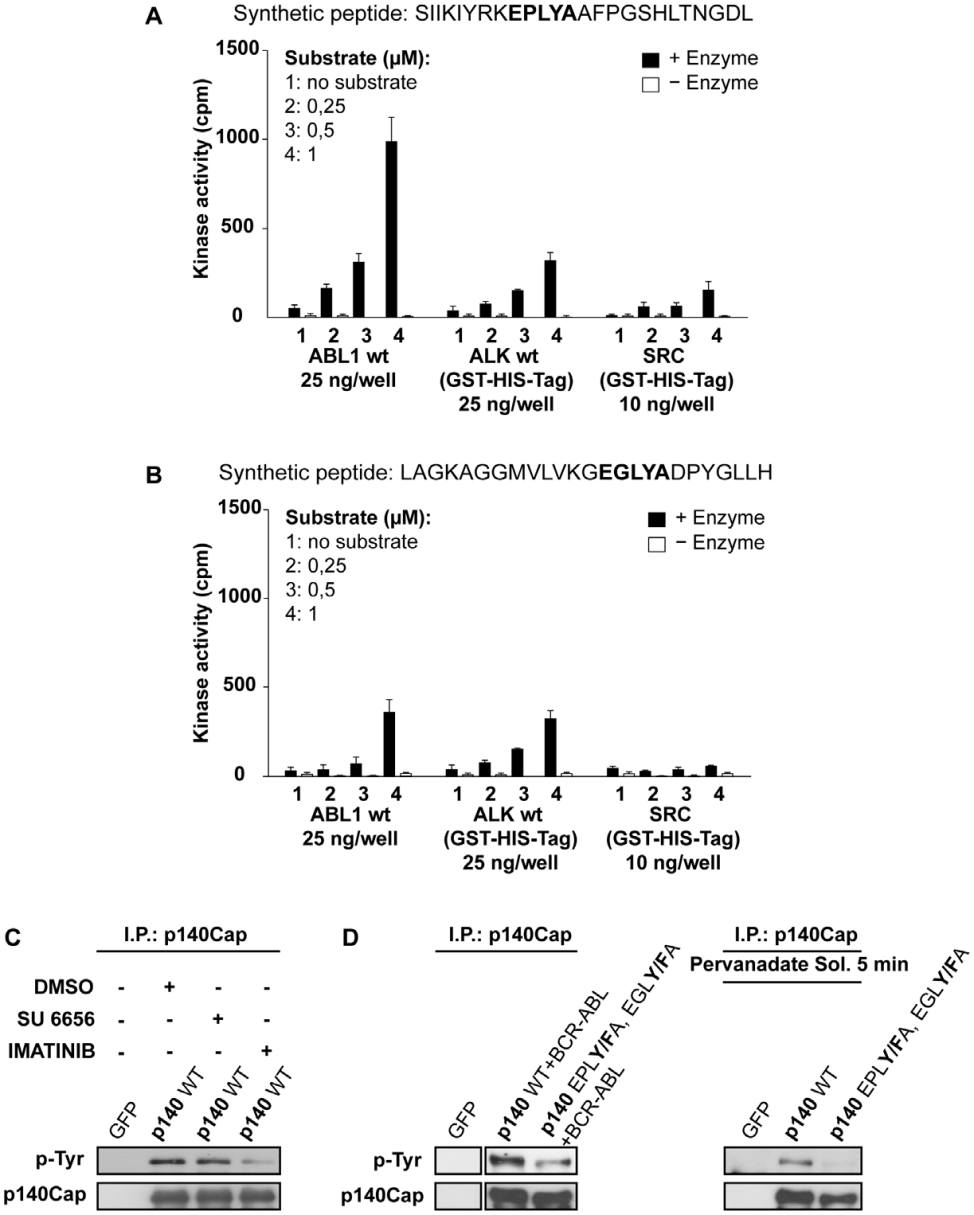
Identification of Abl as the tyrosine kinase responsible of p140Cap tyrosine phosphorylation on EPLYA and EGLYA sequences. A–B. For each recombinant kinase, bar charts of the mean values of the triplicate activity raw counts of kinase activity and the means of the corresponding background values of the synthetic peptides with (black bars) or without (white bars) enzyme are indicated. The synthetic peptides contain respectively EPLYA (A) and EGLYA (B) sequences. A. cDNAs encoding GFP and GFP-p140Cap full length (p140 WT) were used to transfect HEK-293 cells. After 24 hours, cells were starved and treated with 10 micromolar Src inhibitor SU6656 or Abl inhibitor Imatinib for 16 hours. Cell extracts were immunoprecipitated with a specific antibody to p140Cap and analysed by western blotting using monoclonal antibodies for phosphotyrosine and p140Cap. **B.** Left panel. cDNAs encoding GFP, GFP-p140Cap full length (p140 WT) and its double mutant (p140 EPLY/FA, EGLY/FA) were used to transfect HEK-293 cells together with cDNA encoding for active BCR-Abl. Extracts were immunoprecipitated with a specific antibody to p140Cap and analysed by western blotting using monoclonal antibodies to phosphotyrosines (PY99) and p140Cap. Right panel. cDNAs encoding GFP, GFP-p140Cap full length (p140 WT) and its double mutant (p140 EPLY/FA, EGLY/FA) were used to transfect HEK-293 cells. Cells were treated with 100 micromolar pervanadate solution for five minutes and extracts were processed as in the left panel.

In order to identify the tyrosine kinases involved in phosphorylation of the EPLYA and EGLYA sequences, we first performed *in silico* analysis. Based on the fact that three different tyrosine kinases, namely Abl, Alk, and Src displayed the highest scores (data not shown), we took advantage of the KinaseFinder service package (see Materials and Methods) to find out whether the EPLYA and EGLYA peptides could function as substrates for these three recombinant protein tyrosine kinases in an *in vitro* system. As shown in Figure 5A, when different amounts of the synthetic peptide SIIKIYRK**EPLYA**AFPGSHLTNGDL including the EPLYA sequence, were incubated with recombinant purified kinases, only the Abl kinase was able to significantly trigger peptide phosphorylation at both 0,5 and 1 micromolar substrate concentrations. On the other hand, both Abl and Alk kinases were able to phosphorylate the synthetic peptide LAGKAGGMVLVKG**EGLYA**DPYGLLH, including the EGLYA sequence, although with low efficiency, at the high substrate concentration (1 micromolar) (Figure 5B). Since Src kinase was almost inactive in these *in vitro* assays and the ALK kinase is not expressed in HEK-293 cells (data not shown), we focused our analysis on the ability of Abl to trigger p140Cap tyrosine phosphorylation in these cells.

Abl tyrosine kinase is involved in many key cellular processes such as cytoskeleton remodelling, cell shape and movement, through phosphorylation of specific substrates including many adaptor proteins [23], [24]. Notably, interfering with Abl kinase activity by treating HEK-293 cells with 10 micromolar Imatinib, a specific Abl inhibitor [25], resulted in a consistent loss of p140Cap tyrosine phosphorylation (Figure 5C). As already suggested by the *in vitro* kinase assays, the use of Src inhibitor SU6656 did not affect p140Cap tyrosine phosphorylation (Figure 5C). To further assessing the relevance of Abl in p140Cap phosphorylation, HEK-293 cells were transfected with both p140Cap WT and shRNA for Abl silencing (shG10), resulting in down-regulation of Abl expression of about 40%. Upon Abl silencing, p140Cap tyrosine phosphorylation was reduced in a corresponding manner (Supplementary Figure S2). Moreover HEK-293 cells were transiently co-transfected with either p140Cap WT or the double mutant (EPL**Y/F**A, EGL**Y/F**A) together with the constitutive active form of Abl, namely BCR-Abl [25]. The expression of the active BCR-Abl induced p140Cap WT tyrosine phosphorylation (Figure 5D, left panel), at a significantly higher level of that induced upon cell treatment with 100 micromolar pervanadate solution (Figure 5D, right panel). Remarkably, the p140Cap double mutant (EPL**Y/F**A, EGL**Y/F**A) was poorly phosphorylated by active BCR-Abl (Figure 5D, left panel). Taken together, these data demonstrate that both in *in vitro* kinase assays and in HEK293 cells, p140Cap undergoes tyrosine phosphorylation on the EPLYA and EGLYA sequences preferentially through Abl kinase activity.

## Discussion

In this study we provide evidences of p140Cap *in vivo* phosphorylation on one tyrosine and three serine residues in MCF7 breast cancer cells. The *in vivo* tyrosine phosphorylation detected on the EGLYA sequence was validated by site specific mutagenesis, showing that this tyrosine, together with that embedded in the sequence EPLYA, represent the major residues involved in p140Cap phosphorylation. Moreover the EPLYA and EGLYA motifs are also responsible for the interaction between phosphorylated p140Cap and the Csk kinase. Furthermore, we identify Abl as the major tyrosine kinase that can trigger p140Cap phosphorylation on these sequences. Overall these data represent the first attempt to decipher the function of p140Cap post-translational modification, mostly tyrosine phosphorylation, the structural basis of p140Cap-Csk interaction, and one major tyrosine kinase involved in p140Cap phosphorylation.

Phosphorylation on specific amino acid residues leads to conformational changes that modulate and control intrinsic biological activity, sub cellular location, stability, and interaction with other proteins. p140Cap contains many residues that upon phosphorylation could promote protein–protein interactions, leading to the assembly of signalling complexes [5]. Here we show that indeed p140Cap is phosphorylated *in vivo* on three serine residues that are highly conserved and distributed over the entire length of the p140Cap protein. In particular a serine phosphorylated peptide RFpSNVGLVHTSER lies in the N-terminal domain of the protein, while the RGpSDELTVPR and TEKPSKpSPPPPPPR sequences are located in the most C-terminal part, namely in the second proline rich region of p140Cap. Interestingly, p140Cap has already been shown to be phosphorylated on the serine RGpSDELTVPR in a global phospho-proteomic analysis of human and mouse brain extracts [2], [26], [27], suggesting that this serine might play a key role in p140Cap biology. Site specific mutagenesis and the identification of putative binding modules for these phosphorylated serine residues will offer new paradigms for understanding how cell signalling can be regulated by p140Cap serine phosphorylation.

The tyrosine embedded in the EGLYA sequence (GEGLpYADPYGLLHEGR) is phosphorylated *in vivo* and has the highest score of phosphorylation prediction (0.981), based on the use of the NetPhos algorithm [22] indicating that *in silico* data match accurately with the *in vivo* analysis. Moreover, this tyrosine residue has also been identified as phosphorylated by a global phosphoproteomic analysis of murine brain [16]. Interestingly, among the 24 tyrosines present in the p140Cap sequence, only another residue, the tyrosine included in the 261–278 peptide (namely EPLYA), showed a similar level of phosphorylation during an *in silico* prediction (0.980), leading us to analyse the relevance of both residues by site specific mutagenesis. The observation that p140Cap mutated in EPLYA or EGLYA is still phosphorylated on tyrosine even though at a low level, suggests that each of these two tyrosines can be independently phosphorylated. Moreover, the fact that the double mutant in EPLYA and EGLYA is no longer phosphorylated, demonstrates that these two tyrosines are the most relevant in determining p140Cap tyrosine phosphorylation. Regarding the dynamics of the EPLYA modification in human breast cancer MCF7 cells, liquid chromatography data indicated that the tyrosine 264 residue (EPLYA) could be in-vivo phosphorylated in a very dynamic way, with a kinetics different from the tyrosine 396 (EGLYA).

Notably, the tyrosine residues in the sequence FYELE and in the sequence ADPYG - which is very close to the EGLYA and included in the *in vivo* phosphorylated GEGLpYADPYGLLHEGR - do not account for p140Cap phosphorylation. Indeed, while both EGLYA and EPLYA have been both found phosphorylated in human breast cancer cells (http://www.phosphosite.org/), FYELE and ADPYG were not found phosphorylated in human samples. Overall, these results represent the first characterisation of the most significant p140Cap phosphotyrosines in the human setting.

Csk kinase is a potent negative regulator of Src, due to its ability to phosphorylate the negative regulatory tyrosine 527 on the C-terminal domain of Src [28]. Our previous data have already demonstrated that upon adhesion to fibronectin, p140Cap up-regulates Csk activity, leading to increased phosphorylation of Src on tyrosine 527. Moreover, we have also proved that the Csk interacts with p140Cap by Far Western analysis [6]. In this study we show that p140Cap directly interacts with Csk SH2 domain, and that the tyrosine phosphorylation of p140Cap modulates its binding to Csk. Moreover, we identify the phosphorylation of the EGLYA and EPLYA tyrosines as the most relevant for Csk association, either upon pervanadate treatment or physiological FBS stimulus. Taken together, our data imply that the functional interaction between p140Cap and Csk relies on the ability of phosphorylated tyrosine embedded in EGLYA and EPLYA to associate Csk. Moreover, the two sequences EGLYA and EPLYA are similar to the EPIYA motif, that has been found in CagA, an effector protein involved in Helicobacter Pylori pathogenesis [17]–[20]. The bacterial CagA protein contains several (from one to five) repeated EPIYA or EPIYA-like sequences that, upon delivery into mammalian cells, undergo tyrosine phosphorylation, leading to pathogenesis through the formation of complexes with SH2 domain-containing proteins. Remarkably, in Helicobacter pylori the CagA EPIYA sequence is involved in binding of Csk SH2 domain, resulting in Csk membrane recruitment with subsequent inhibition of SFKs [19], [20]. Interestingly, the EPIYA motif has similar functions in the mammalian Pragmin/SgK223 protein, as a module able to interact with the Csk SH2 domain. An elevated SFK activity is detected in cells expressing Pragmin, collectively indicating that this protein provokes cell morphological transformation by sequestering Csk and potentiating SFK kinase activity [21]. Thus, expression of proteins like Pragmin or p140Cap that contain EPIYA-like motifs could interfere with Csk activation [28] and/or localisation [21], finely tuning SFK activity inside the cells.

We have shown here that the EPLYA and the EGLYA sequences are relevant for both vanadate- and serum-dependent phosphorylation, indicating that these tyrosine residues might be substrate of several tyrosine kinases. Our data provide evidence that p140Cap is the substrate for the Abl kinase. Abl is an ubiquitous tyrosine kinase that controls actin remodelling, cell motility and adhesion, and is involved in cell differentiation processes through association with specific substrates, most of which mediate signal transduction and cytoskeleton dynamics [23], [24]. Indeed *in silico* analysis coupled to *in vitro* kinase assays with recombinant tyrosine kinases allowed the identification of Abl as the major kinase involved in tyrosine phosphorylation of EPLYA and EGLYA synthetic peptides. Consistently, in HEK-293 cells, the specific Abl inhibitor Imatinib strongly reduces p140Cap tyrosine phosphorylation. Moreover, in the same cellular system, tyrosine phosphorylation triggered by the constitutive active BCR-Abl kinase was heavily reduced on the double p140Cap mutant (EPLY/FA, EGLY/FA). ABL specificity for a given substrate is conferred by both target sequence and domain-guided protein-protein interactions. Notably, the EPLYA and EGLYA sequences fit well with the Abl target site consensus sequence [23]. In particular, they contain aliphatic amino acids (L) at position –1, as well as acidic residues (E) at positions –3, thus ascribing p140Cap as a new Abl kinase substrate. Interestingly, our data are further supported by recent reports showing that Abl is involved in EPIYA repeats phosphorylation in different CagA strains infected cells and that phosphorylation of these sites controls cell elongation upon CagA infection [29], [30].

In conclusion, we identified serine and tyrosine phosphorylated residues on the human p140Cap adaptor in breast cancer cells. Moreover we validated two tyrosine residues inserted in the EGLYA and EPLYA sequences as the major phosphorylated sites responsible for p140Cap tyrosine phosphorylation and Csk kinase binding. We also found that p140Cap phosphorylation on the EPLYA and EGLYA sequences in HEK-293 cells is dependent on the Abl tyrosine kinase activity. Overall, elucidating the role of the EGLYA and ELPYA sequences in p140Cap may give further insights into the mechanisms underlying p140Cap biological activity in both physiological and pathological conditions.

## Supporting Information

Figure S1 **Expression of p140Cap EPLYA/EGLYA double mutant partially rescues Src activation.** cDNAs encoding GFP, GFP-p140Cap WT and GFP-p140Cap EPLYA/EGLYA double mutant were used to transfect HEK-293 cells. After 48 hours, cells were starved overnight and treated with 20% FBS for the indicated times. Cell extracts were western blotted using polyclonal antibodies to p140Cap, active Src (pY416) and total Src. The results are representative of two independent experiments. Numbers express the quantification of the ratio between active Src and total Src, with 100 as arbitrary value assigned to the levels of Src phosphorylation in GFP-transfected cells.
(PSD)

Figure S2 **Abl silencing in HEK-293 cells causes down-regulation of p140Cap tyrosine phosphorylation.** cDNA encoding GFP-p140Cap WT was used to transfect HEK-293 cells together with Abl shRNA construct G10. After 48 hours, cell extracts were immunoprecipitated with a specific antibody to p140Cap and analysed by western blotting using monoclonal antibodies to phosphotyrosine PY99 and p140Cap. Abl silencing was evaluated on cell extracts. The results are representative of two independent experiments.
(PSD)
